# Morphokinetic Markers of Blastocyst Formation and Chromosomal Euploidy in Embryos Derived From In Vitro Maturation Oocytes

**DOI:** 10.1002/rmb2.70037

**Published:** 2026-03-30

**Authors:** Hong Chen, Ruolin Mao, Rui Long, Juepu Zhou, Huijun Li, Qingsong Xi, Lixia Zhu, Lei Jin

**Affiliations:** ^1^ Reproductive Medicine Center, Tongji Hospital, Tongji Medical College Huazhong University of Science and Technology Wuhan China; ^2^ Laboratory Medicine Department, Tongji Hospital, Tongji Medical College Huazhong University of Science and Technology Wuhan China; ^3^ Oncology Center, Tongji Hospital, Tongji Medical College Huazhong University of Science and Technology Wuhan China

**Keywords:** embryo development, euploidy, in vitro maturation, morphokinetics, time‐lapse imaging

## Abstract

**Purpose:**

To characterize the morphokinetic parameters of embryos derived from IVM oocytes and to identify critical factors associated with blastocyst formation potential and chromosomal euploidy.

**Methods:**

This retrospective study reviewed 99 523 fresh cycles between January 2019 and March 2025. From this population, 3,202 PGT cycles were initially screened. A total of 353 cycles involving IVM oocytes were then selected, and ultimately, 83 IVM‐derived embryos from 59 cycles were included.

**Results:**

The non‐formation group exhibited delays in t2, t9, and several developmental intervals, including S2, t9–t7, t9–t8 (*p* < 0.05). Multivariate logistic regression analysis identified S2 (*p* = 0.021) and t9–t7 (*p* = 0.042) as independent factors associated with blastocyst formation. Subsequently, ROC analysis showed that both S2 ≤ 1.95 h (AUC = 0.620, *p* = 0.036) and t9–t7 ≤ 24.35 h (AUC = 0.672, *p* < 0.001) exhibited predictive power for blastocyst formation. Furthermore, shorter tSB–t9 and tB–t9 were associated with euploidy in IVM‐derived blastocysts (*p* = 0.025 and *p* = 0.014, respectively), with tSB–t9 emerging as an independent factor associated with chromosomal normality (*p =* 0.044) in multivariate analysis.

**Conclusions:**

This study presents the morphokinetic characteristics and identifies key markers for blastocyst formation and chromosomal euploidy in IVM‐derived embryos.

## 1 Introduction

In vitro maturation (IVM) has become a key strategy in assisted reproductive technology, particularly for patients with diminished ovarian reserve (DOR), polycystic ovary syndrome (PCOS), or those at high risk of ovarian hyperstimulation syndrome (OHSS) [1, 2, 3]. IVM allows for the retrieval and in vitro maturation of immature oocytes (up to 30% in conventional cycles), bypassing hormonal risks and improving oocyte maturation [4, 5, 6]. This strategy increases the number of viable embryos, enhances cumulative live birth rates, and offers a valuable alternative for patients unable to obtain mature oocytes through conventional stimulation [7, 8].

However, studies demonstrate that IVM‐derived oocytes and embryos exhibit reduced developmental competence, including lower fertilization rates, delayed cleavage kinetics, and decreased blastocyst formation rates, compared to those derived from in vivo‐matured oocytes [9, 10]. These differences may arise from incomplete cytoplasmic maturation, abnormal epigenetic modifications, or suboptimal in vitro culture conditions, all of which can affect embryo quality and increase the risk of chromosomal abnormalities [11, 12]. Consequently, invasive techniques such as preimplantation genetic testing (PGT) are commonly used to screen embryos for chromosomal abnormalities, such as aneuploidy. Although PGT is widely used to screen for chromosomal abnormalities, its invasiveness, high cost, and inability to reliably detect mosaicism highlight the need for non‐invasive selection tools, especially for IVM embryos [13, 14].

Time‐lapse imaging (TLI) offers a promising alternative for non‐invasive embryo assessment by continuously monitoring development without disrupting culture conditions [15]. Specific morphokinetic parameters, such as time to pronuclear fading (tPNf), cleavage synchrony (S2/S3), and time to blastocyst formation (tB), are strongly associated with embryo potential, implantation, and chromosomal normality in conventional IVF cycles [16, 17, 18, 19, 20]. Yet, IVM‐derived embryos exhibit distinct developmental trajectories, such as delayed cleavage, asynchronous cell cycles, and delayed blastocyst formation, rendering IVF‐based TLI criteria suboptimal [9, 21, 22]. This discrepancy is further complicated by inconsistent findings in existing research: some note that pronuclear fading and early cleavage are delayed in IVM‐derived embryos [9], while others report accelerated development during the pronuclear stage followed by delays after the 6‐cell stage [23]. Such inconsistencies highlight the lack of standardized morphokinetic profiles for IVM embryos. Therefore, there is an urgent need to optimize morphokinetic parameters for IVM embryos to enhance the accuracy of embryo assessment and ART outcomes. Currently, IVM‐specific morphokinetic benchmarks remain undefined, and no studies simultaneously evaluate their association with blastulation and euploidy.

This study aims to characterize the morphokinetic parameters of IVM‐derived embryos and identify key indicators that can reliably predict blastocyst formation potential and chromosomal normality. By identifying these parameters, we hope to establish non‐invasive biomarkers based on time‐lapse imaging to optimize embryo selection strategies in IVM cycles.

## 2 Methods

### 2.1 Study Participants

**FIGURE 1:**
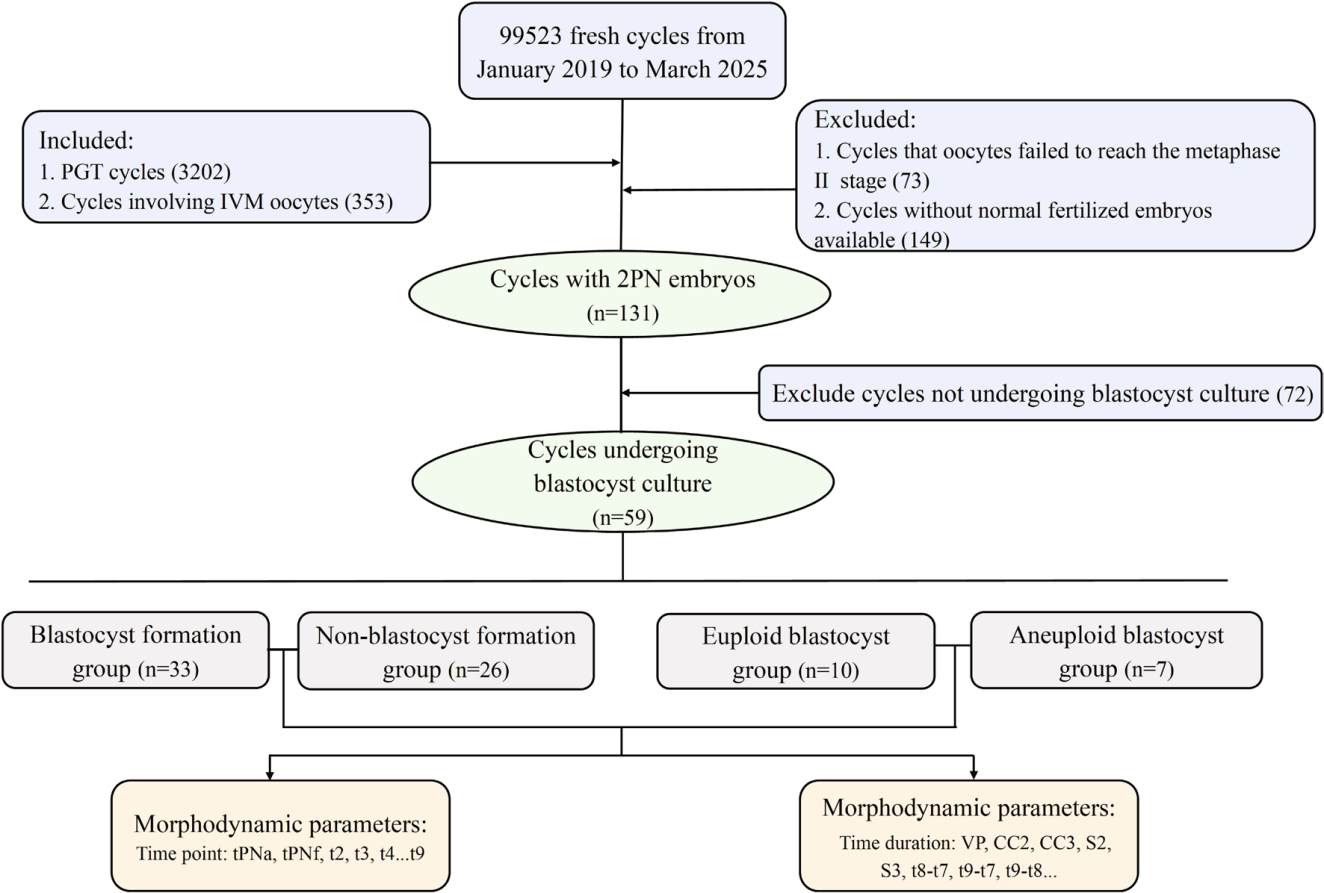
Data collection and analysis flowchart. Among the blastocysts formed, ploidy analysis was performed only on blastocysts with sufficient morphological grading and with patient consent. Numbers shown in the flowchart represent cycles, whereas subsequent analyses were performed at the embryo level. 2PN, two pronuclei; IVM, in vitro maturation; PGT, preimplantation genetic testing; tPNa, time to pronuclear appearance; tPNf, time of pronuclear fading; tn (*n* = 2–9): Time to *n* cells; VP, tPNf–tPNa; CC2, t3–t2; CC3, t5–t3; S2, t4–t3; S3, t8–t5.

This retrospective study initially included 99 523 fresh assisted reproductive technology (ART) cycles performed at our center from January 2019 to March 2025. From these cycles, 3202 PGT cycles were selected, and 353 cycles involving IVM oocytes were further selected from the PGT cycles. Further exclusion criteria included: (1) cycles in which oocytes failed to reach metaphase II stage, (2) cycles without normal fertilized embryos available, and (3) cycles not subjected to blastocyst culture. Finally, 83 IVM‐derived embryos from 59 PGT cycles were analyzed (Figure 1). These embryos were categorized into two groups based on their ability to form blastocysts: blastocyst formation group and non‐blastocyst formation group. Additionally, based on their chromosomal status after blastocyst biopsy, the embryos were further classified into euploid blastocyst and aneuploid blastocyst groups. The relationship between morphokinetic parameters and embryo development and quality was analyzed between the groups, aiming to assess the predictive value of these parameters in distinguishing the groups.

### 2.2 Oocyte Retrieval and In Vitro Maturation

Standardized ovarian stimulation protocols (GnRH agonist or GnRH antagonist) were employed, as described in previous literature [24]. The stimulation protocol was tailored to each patient's individual characteristics. When ≥ 2 follicles reached 18 mm in diameter, recombinant hCG (Ovidrel, Merck Serono) was administered to trigger ovulation. Oocyte retrieval was performed 36 h later under transvaginal ultrasound guidance. Immature oocytes at germinal vesicle (GV) or metaphase I (MI) stages were collected and cultured in pre‐equilibrated G1‐plus medium (Vitrolife, Sweden) in an EmbryoScope Plus time‐lapse incubator (Vitrolife, Sweden) under 37°C, 6% CO_2_, and 5% O_2_ conditions. Maturation was indicated by the extrusion of the first polar body (MII), and these oocytes were then used for fertilization.

### 2.3 Fertilization and Embryo Culture

Mature oocytes were fertilized via intracytoplasmic sperm injection (ICSI) using sperm processed by density gradient centrifugation (SpermGrad, Vitrolife), following established procedures [25]. Fertilization was assessed 16–18 h post‐ICSI by confirming the presence of two pronuclei (2PN) and two polar bodies. Fertilized embryos were cultured in an EmbryoScope Plus incubator using a sequential culture system: G1‐plus medium (Vitrolife, Sweden) for Days 1–3, and G2‐plus medium (Vitrolife, Sweden) for Days 3–5/6, maintaining constant conditions of 37°C, 6% CO_2_, and 5% O_2_. Blastocyst formation was defined by the appearance of an expanded blastocyst cavity, a clear inner cell mass (ICM), and a trophoblast layer (TE), and embryo quality was assessed according to the Gardner grading system.

### 2.4 Time‐Lapse Imaging and Morphokinetic Parameters

**FIGURE 2:**
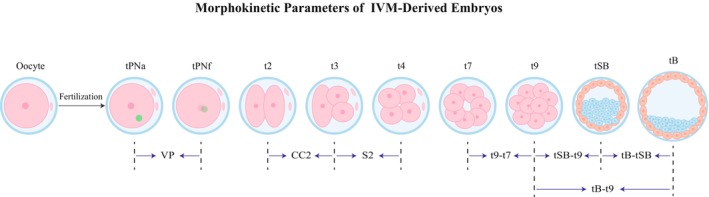
Schematic diagram of morphokinetic parameters. IVM, in vitro maturation; tPNa, time to pronuclear appearance; tPNf, time of pronuclear fading; tn: Time to *n* cells; tSB, time to start of blastulation; tB, time to blastocyst formation; VP, tPNf–tPNa; CC2, t3–t2; S2, t4–t3.

Embryo development from fertilization to the blastocyst stage was dynamically monitored using the EmbryoScope Plus system, which captured images every 10 min across 11 focal planes with an inter‐plane distance of 150 μm. Morphokinetic parameters were annotated by two senior embryologists, each with over 5 years of experience in embryology, and the annotation process was blinded to the PGT results. Both embryologists had undergone internal standardization training to ensure consistent annotation. In cases of ambiguity, a consensus was reached by discussion. The recorded parameters included: (1) time‐point parameters: tPNa (time of pronuclear appearance), tPNf (time of pronuclear fading), tn (*n* = 2–9, time to *n* cells), tSB (time to start of blastulation, initial blastocyst cavity formation), and tB (time to blastocyst formation, complete blastocyst expansion); (2) interval parameters: VP (duration of pronuclear existence, tPNf–tPNa), CC2 (interval between 2 and 3 cells, t3–t2), CC3 (interval between 3 and 5 cells, t5–t3), S2 (second synchrony, interval between 3 and 4 cells, t4–t3), S3 (third synchrony, interval between 5 and 8 cells, t8–t5), t9–t7 (interval between 7 and 9 cells), t9–t8 (interval between 8 and 9 cells), tSB–t9 (interval from 9 cells to start of blastulation), and tB–t9 (interval from 9 cells to blastocyst formation) [26]. Time zero (t0) was defined as the time of ICSI. All morphokinetic time points and intervals were calculated relative to t0. A schematic overview of these parameters in relation to embryonic development is provided in Figure 2.

### 2.5 Preimplantation Genetic Testing

PGT was performed using next‐generation sequencing (NGS) technology. Trophectoderm biopsy was conducted on blastocysts when clinically indicated, primarily based on blastocyst morphological grading and patient consent, and the collected cells underwent whole‐genome amplification (WGA). Libraries were sequenced on the Life Technologies Ion Proton platform at ~0.04× genome depth to analyze copy number variations (CNVs) across all 24 chromosomes (1–22, X, Y). Embryos were classified as euploid if they met the following criteria: (1) normal copy number of all chromosomes; (2) absence of segmental deletions/duplications ≥ 5 Mb; (3) mosaicism ratios below the chromosomal‐specific thresholds (chromosomes 13/16/18/21: < 30%; chromosome 19: < 50%; others: < 40%) [27].

### 2.6 Statistical Analysis

Statistical analysis was performed using SPSS 27.0. Continuous variables were tested for normality using the Shapiro–Wilk test. Normally distributed data were expressed as mean ± standard deviation (SD), and non‐normally distributed data as median (interquartile range, IQR). Categorical variables were presented as number (percentage). Intergroup comparisons were initially performed using the Mann–Whitney *U* test. Univariate logistic regression analysis was used to identify morphokinetic parameters associated with outcomes including blastocyst formation and euploidy, and multivariate logistic regression was employed to identify independent predictors. Before performing multivariate analysis, multicollinearity among candidate predictors was assessed using the variance inflation factor (VIF) and tolerance. A VIF < 5 (tolerance > 0.2) was set as the acceptable threshold. Receiver operating characteristic (ROC) curves were used to evaluate the predictive performance of significant parameters, with the optimal cutoff determined by the Youden index. To internally validate the predictive models and correct for optimism bias, we performed bootstrap resampling. To account for potential within‐patient correlations, a sensitivity analysis using a mixed‐effects logistic regression model with patient ID as a random intercept was also performed for the outcome of blastocyst formation. Statistical significance was defined as a two‐tailed *p* < 0.05.

## 3 Results

### 3.1 Baseline Characteristics of the Study Cohort

This retrospective study included 83 embryos derived from IVM across 59 PGT cycles. The baseline characteristics of the patients are summarized in Table 1. The mean age of the patients was 31.3 ± 4.7 years, and the mean body mass index (BMI) was 22.5 ± 3.0 kg/m^2^. Secondary infertility accounted for 76.3% of the cases, with a median infertility duration of 1.5 years. In addition, Table 1 also details additional baseline characteristics, including ovarian reserve, ovarian stimulation, and ovarian response.

**TABLE 1:** Baseline characteristics of IVM‐derived embryo cycles (*n* = 59).

Characteristics	Values
Age, years	31.3 ± 4.7
BMI, kg/m^2^	22.5 ± 3.0
Infertility type, *n* (%)	
Primary	14 (23.7%)
Secondary	45 (76.3%)
Infertility duration, years	1.5 (1.0–2.0)
Basal serum FSH level, mIU/mL	7.1 (6.2–8.2)
Serum AMH level, ng/mL	3.9 (2.2–5.5)
Antral follicle count	12.0 (8.0–21.0)
Ovarian stimulation protocol, *n* (%)	
GnRH agonist protocol	29 (49.2%)
GnRH antagonist protocol	30 (50.8%)
Duration of stimulation, days	10.0 (9.0–11.0)
Gonadotropin dose, IU	2538.9 ± 868.9
Ovarian response	
Serum E2 level on hCG trigger day, pg/mL	2228.0 (1707.0–3444.0)
Serum P level on hCG trigger day, ng/mL	0.8 (0.5–1.3)
Oocytes retrieved, *n*	13.0 (9.0–17.0)
MII oocytes retrieved, *n*	9.0 (7.0–14.0)
Maturation rate at retrieval	76.6%
Normal fertilization rate	76.7%
PGT cycles, *n* (%)	
PGT‐A	24 (40.7%)
PGT‐M	3 (5.1%)
PGT‐SR	32 (54.2%)

*Note:* Based on whether the data follow a normal distribution, continuous values were described as mean ± SD or median (interquartile range).

Abbreviations: AMH, anti‐Müllerian hormone; BMI, body mass index; E2, estradiol; FSH, follicle stimulating hormone; GnRH, gonadotrophin releasing hormone; P, progesterone; PGT, preimplantation genetic testing; PGT‐A, PGT for aneuploidy; PGT‐M, PGT for monogenic disorders; PGT‐SR, PGT for structural chromosomal rearrangements.

### 3.2 Morphokinetic Markers for Blastocyst Formation

**FIGURE 3:**
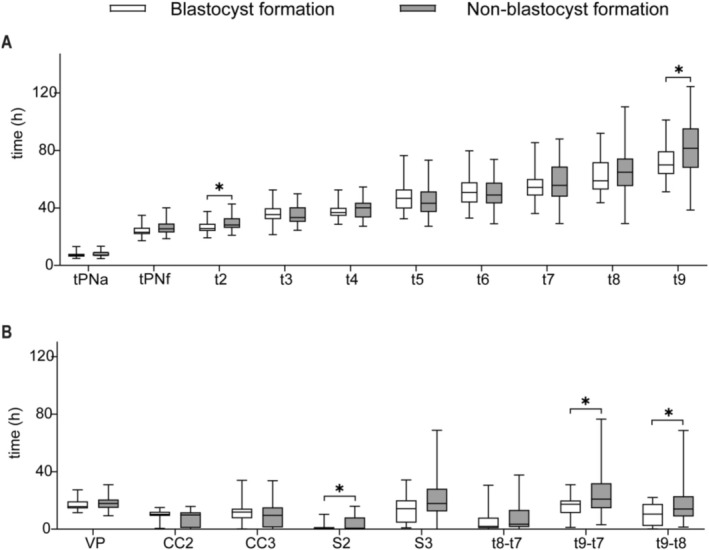
Comparison of morphokinetic parameters between blastocyst formation and non‐formation groups. (A) Box plots depicting morphokinetic times (in hours after insemination). (B) Box plots depicting morphokinetic durations (in hours). Data are shown as box plots, where the horizontal line in the box represents the median, the top and bottom of box represent the 75th and 25th quartiles, respectively, and the upper and lower whiskers represent the maximum and minimum, respectively. h, hour; tPNa, time to pronuclear appearance; tPNf, time to pronuclear fading; tn (*n* = 2–9): Time to *n* cells; VP, tPNf‐tPNa; CC2, t3–t2; CC3, t5–t3; S2, t4–t3; S3, t8–t5. Significant differences (*p < 0.05) are indicated between the marked groups by the Mann–Whitney *U* test.

Significant differences in morphokinetic parameters were observed between the blastocyst formation and non‐formation groups. The non‐formation group exhibited substantial developmental delays, including delayed early cleavage time (t2) (28.2 h vs. 25.6 h; *p* = 0.024), delayed late developmental milestone (t9) (81.5 h vs. 70.0 h; *p* = 0.024), longer synchrony index S2 interval (0.7 h vs. 0.5 h; *p* = 0.050), as well as extended time intervals t9–t7 (20.9 h vs. 17.4 h; *p* = 0.012) and t9–t8 (14.1 h vs. 10.5 h; *p* = 0.039) (Figure 3, Table S1). Univariate logistic regression analysis confirmed that these parameters (t2, t9, S2, t9–t7, and t9–t8) were significantly associated with blastocyst formation (Table 2). Collinearity diagnostics for these candidate predictors revealed that all VIF values were well below the threshold of 5 (range: 1.029–3.487); hence, no variables were excluded on multicollinearity grounds. Subsequently, multivariate logistic regression analysis further identified S2 (OR = 1.17; 95% CI: 1.02–1.33; *p* = 0.021) and t9–t7 (OR = 1.09; 95% CI: 1.00–1.18; *p* = 0.042) as independent factors associated with blastocyst formation (Table 3).

**TABLE 2:** Univariate logistic regression analysis of morphokinetic parameters in relation to blastocyst formation.

Parameters (hour)	Blastocyst formation	Non‐blastocyst formation	*p*	OR (95% CI)
t2	25.6 (24.0–29.1)	28.2 (26.1–32.9)	0.033*	1.12 (1.01–1.24)
t9	70.0 (63.6–79.4)	81.5 (67.9–95.4)	0.026*	1.04 (1.00–1.07)
S2	0.5 (0.2–1.5)	0.7 (0.3–8.3)	0.032*	1.13 (1.01–1.26)
t9–t7	17.4 (11.2–20.1)	20.9 (14.6–32.0)	0.005*	1.09 (1.03–1.16)
t9–t8	10.5 (2.3–17.6)	14.1 (8.8–22.9)	0.014*	1.07 (1.01–1.14)

*Note:* Parameters were described as median (interquartile range). tn: time to *n* cells; S2, t4–t3.

* *p* < 0.05.

**TABLE 3:** Multivariate logistic regression analysis for blastocyst formation.

Parameters (hour)	*p*	OR (95% CI)
t2	0.215	1.07 (0.96–1.19)
t9	0.795	0.99 (0.95–1.04)
S2	0.021*	1.17 (1.02–1.33)
t9–t7	0.042*	1.09 (1.00–1.18)
t9–t8	0.691	1.02 (0.94–1.10)

*Note:* Multivariate logistic regression analysis based on collinearity diagnosis. tn: time to *n* cells; S2, t4–t3.

* *p* < 0.05.

Subsequently, ROC curve analysis was performed to evaluate the predictive performance of these parameters (Table 4). The optimal cut‐off values determined by the Youden index were S2 ≤ 1.95 h (AUC = 0.620, *p* = 0.036) and t9–t7 ≤ 24.35 h (AUC = 0.672, *p* < 0.001). Embryos meeting the S2 ≤ 1.95 h criterion demonstrated a significantly higher blastocyst formation rate (62.7%) compared to those exceeding this threshold (37.5%; *p* < 0.05). Similarly, embryos with t9–t7 ≤ 24.35 h showed a markedly increased formation rate (72.6%) than those with t9–t7 > 24.35 h (7.1%; *p* < 0.001). Furthermore, t2 ≤ 26.1 h (AUC = 0.645, *p* = 0.003), t9 ≤ 82.3 h (AUC = 0.654, *p* = 0.001), and t9–t8 ≤ 22.45 h (AUC = 0.641, *p* < 0.001) also demonstrated predictive value for blastocyst formation. To internally validate these models and correct for optimism, bootstrap resampling was applied. The bootstrap‐corrected AUC for the S2 (≤ 1.95 h) model was 0.623 (95% CI: 0.503–0.749), with an optimism of −0.003. For the t9–t7 (≤ 24.35 h) model, the corrected AUC was 0.668 (95% CI: 0.537–0.788), with an optimism of 0.004.

**TABLE 4:** ROC analysis and binary classification performance of blastocyst formation predictors.

Parameters (hour)	AUC	Cut‐off value	Binary classification	Blastocyst formation rate	*p*	OR (95% CI)	Sensitivity	Specificity	PPV (%)	NPV (%)
t2	0.645	26.1	≤ 26.1	74.3% (26/35)	0.003*	4.04 (1.56–10.47)	0.565	0.757	74.3	58.3
			> 26.1	41.7% (20/48)						
t9	0.654	82.3	≤ 82.3	72.2% (39/54)	0.001*	5.57 (1.90–16.35)	0.848	0.500	72.2	68.2
			> 82.3	31.8% (7/22)						
S2	0.62	1.95	≤ 1.95	62.7% (37/59)	0.036*	2.80 (1.05–7.47)	0.804	0.405	62.7	62.5
			> 1.95	37.5% (9/24)						
t9–t7	0.672	24.35	≤ 24.35	72.6% (45/62)	< 0.001*	34.41 (4.18–283.58)	0.978	0.433	72.6	92.9
			> 24.35	7.1% (1/14)						
t9–t8	0.641	22.45	≤ 22.45	67.6% (46/68)	< 0.001*	—	1	0.267	67.6	100.0
			> 22.45	0 (0/8)						

*Note:* Cut‐off value determined by Youden's index; Blastocyst formation rate shown as percentage (*n*/*N*) and compared using Chi square test or Fisher's exact test. For t9–t8 > 22.45 h, no blastocysts formed (0/8), leading to complete separation; therefore, OR is not estimable (—) and sensitivity equals 1. tn: time to *n* cells; S2, t4–t3.

Abbreviations: AUC, area under the curve; NPV, negative predictive value; PPV, positive predictive value.

* *p* < 0.05.

A sensitivity analysis employing a mixed‐effects logistic regression model, which accounted for within‐patient clustering by specifying patient ID as a random intercept, was conducted to assess the robustness of the primary findings. This analysis confirmed that the associations of both S2 (*p* = 0.035) and t9–t7 (*p* = 0.012) with blastocyst formation remained statistically significant (Table S3). The variance estimate for the random intercept was not significant (*p* = 0.534), suggesting limited residual clustering effects beyond the explanatory power of these morphokinetic parameters in our cohort.

### 3.3 Morphokinetic Markers for Euploid Blastocysts

**FIGURE 4:**
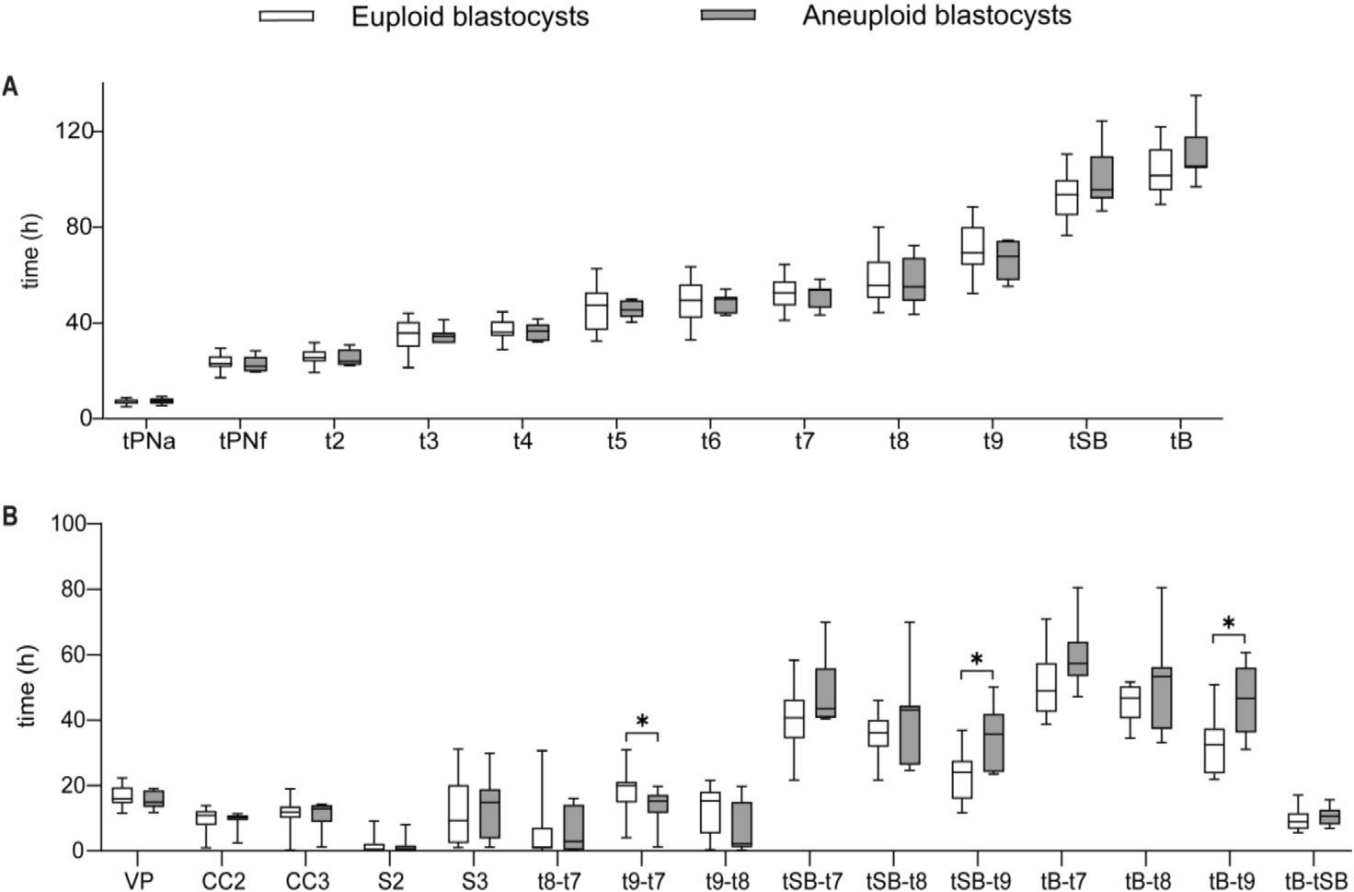
Comparison of morphokinetic parameters between euploid and aneuploid blastocyst groups. (A) Box plots depicting morphokinetic times (in hours after insemination). (B) Box plots depicting morphokinetic durations (in hours). Data are shown as box plots, where the horizontal line in the box represents the median, the top and bottom of the box represent the 75th and 25th quartiles, respectively; the upper and lower whiskers represent the maximum and minimum, respectively. h, hour; tPNa, time to pronuclear appearance; tPNf, time to pronuclear fading; tn (*n* = 2–9): Time to *n* cells; tSB, time to start of blastulation; tB, time to blastocyst formation; VP, tPNf–tPNa; CC2, t3–t2; CC3, t5–t3; S2, t4–t3; S3, t8–t5. Significant differences (*p < 0.05) are indicated between the marked groups by Mann–Whitney – test.

Among the blastocysts that formed, significant differences in specific morphokinetic intervals were observed between euploid and aneuploid groups (Figure 4, Table S2). Euploid blastocysts exhibited significantly shorter intervals of tSB–t9 (24.0 h vs. 35.7 h; *p* = 0.025) and tB–t9 (32.6 h vs. 46.7 h; *p* = 0.014) compared to aneuploid blastocysts (Table S2). Univariate logistic regression analysis confirmed these associations, with tSB–t9 (OR = 1.19, 95% CI: 1.02–1.39, *p* = 0.029) and tB–t9 (OR = 1.15, 95% CI: 1.02–1.31, *p* = 0.028) being significantly associated with euploidy (Table 5). Multivariate logistic regression analysis further identified a significant association between a shorter tSB–t9 interval and euploid blastocysts (OR = 1.17, 95% CI: 1.00–1.36, *p* = 0.044). In contrast, no significant differences were observed between euploid and aneuploid blastocysts for other morphokinetic parameters, such as tPNa, tPNf, or t2–t8 (all *p* > 0.05).

**TABLE 5:** Univariate logistic regression analysis of morphokinetic parameters in relation to euploid blastocysts.

Parameters (hour)	Euploid blastocysts	Aneuploid blastocysts	*p*	OR (95% CI)
t9–t7	20.0 (14.9–21.2)	15.2 (11.6–17.2)	0.175	0.90 (0.78–1.05)
tSB–t9	24.0 (15.9–27.7)	35.7 (24.1–41.9)	0.029*	1.19 (1.02–1.39)
tB–t9	32.6 (23.6–37.5)	46.7 (36.2–56.1)	0.028*	1.15 (1.02–1.31)

*Note:* Parameters were described as median (interquartile range). tn: time to *n* cells.

Abbreviations: tB, time to blastocyst formation; tSB, time to start of blastulation.

* *p* < 0.05.

## 4 Discussion

Previous studies predominantly focused on the morphokinetics of embryo development in conventional IVF, while this study systematically explored the morphokinetic characteristics of IVM‐derived embryos using time‐lapse imaging technology and identified key parameters associated with blastocyst formation and chromosomal euploidy. Our research specifically addresses the unique developmental trajectory of IVM embryos and demonstrates that morphokinetic parameters such as S2, t9–t7, and tSB–t9 serve as reliable markers for evaluating the developmental potential and chromosomal normality of IVM embryos. The present study provided valuable insights for improving IVM‐derived embryo selection in ART.

Blastocyst formation is a critical milestone in embryo development, reflecting the embryo's ability to progress beyond early cleavage stages [28]. For conventional IVF embryos, numerous studies have explored the predictive value of morphokinetic parameters for blastocyst formation, yielding relatively clear predictive indicators: early parameters such as rapid cleavage (shorter t3 and t4) are associated with a higher likelihood of forming high‐quality blastocysts [29]; late parameters like tM and tSB are positively correlated with the speed of blastocyst development [18, 30]; and synchronization parameters such as shorter S2 significantly increase the blastocyst formation rate [31]. However, the developmental morphokinetic parameters of IVM‐derived embryos remain less defined and lack consensus. For instance, Margalit et al. pointed out that early key developmental events in IVM embryos, such as polar body extrusion and pronuclear fading, are significantly delayed [9]. In contrast, Roesner et al. indicated that IVM embryos exhibit accelerated development during the pronuclear stage, only to show a significant slowdown in growth dynamics after reaching the 6‐cell stage [23]. Moreover, current research has primarily focused on comparing the morphokinetic differences between IVM and IVF embryos, with fewer studies addressing the predictive value of morphokinetic parameters specifically in IVM embryos. Against this backdrop, our study enhances the understanding of IVM‐derived embryos by systematically analyzing the entire development process, from fertilization to blastocyst formation, and covering key time points and intervals, in contrast to previous studies that focused on isolated stages or limited parameters. Additionally, we introduced new time intervals not previously explored in the literature, such as t9–t7 and tSB–t9. Our study identified S2 (the synchronization of cleavage during the 3–4 cell stage) and t9–t7 (the time interval between the 7–9 cell stages) as independent factors associated with blastocyst formation in IVM‐derived embryos. Furthermore, we validated the clinical relevance of our findings through ROC analysis, establishing cut‐off values (S2 ≤ 1.95 h, t9–t7 ≤ 24.35 h) that provide actionable metrics for embryo selection. While bootstrap internal validation showed that optimism in model performance remained minimal, supporting the robustness of these thresholds within our cohort, this does not establish their generalizability to other IVM populations. Therefore, these cut‐off values should be considered preliminary. Their definitive clinical utility and broader applicability await prospective validation in independent, external cohorts.

S2 was significantly shorter in embryos that formed blastocysts compared to those that did not, suggesting that a shorter cleavage synchrony interval may be associated with better embryo developmental potential. This finding is consistent with previous research by Margalit et al. which indicated that IVM embryos often exhibit abnormal synchronization, particularly in the second cell cycle, which can impair developmental potential [9]. The underlying mechanism may relate to incomplete cytoplasmic maturation of oocytes during in vitro maturation, which could lead to insufficient accumulation of cell cycle regulators (such as the Cyclin B/CDK1 complex), thereby impairing cleavage coordination and prolonging the S2 phase [12, 32, 33]. Furthermore, research by Yang et al. has shown that prolonged GV‐MI (from germinal vesicle breakdown to metaphase I) duration significantly reduces the developmental potential of IVM oocytes, indicating that maturation‐related dynamics could indirectly lead to synchronization defects in cleavage [25]. Moreover, our multivariate analysis established the independent predictive value of S2 (*p* = 0.021), suggesting it could be a key indicator for early screening of IVM embryos.

The t9–t7 interval is another important predictive parameter. When t9–t7 ≤ 24.35 h, the blastocyst formation rate was as high as 72.6%. The 7–9 cell stage is a critical period during the transition from the cleavage stage to the morula stage and is also the key window for zygotic genome activation (ZGA) [34, 35]. At this stage, embryos are highly dependent on the integrity of their genome. Our findings align with the study by Walls et al. who reported that IVM embryos show a higher early arrest rate compared to in vivo‐matured embryos, especially at the 3–4 day stage [21]. This is consistent with our observation that delayed division at the 7–9 cell stage could indicate genomic abnormalities and may contribute to embryo elimination.

It is noteworthy that our study also found that delays in t2 and t9 were associated with reduced blastocyst formation rates. This aligns with previous studies indicating that IVM embryos commonly exhibit delayed cleavage [9, 36], potentially due to mitochondrial dysfunction in IVM oocytes [37, 38]. Mitochondria are essential for energy metabolism, and their dysfunction can lead to insufficient ATP production, thus delaying cell division [39]. However, multivariate analysis showed that t2 and t9 were not independent predictive factors, suggesting that their influence on blastocyst formation may be modulated by parameters like S2 and t9–t7. This also reflects the complex interplay of multiple developmental stages, where a single time‐point parameter alone may not be a reliable predictor.

Chromosomal euploidy is a critical factor influencing the clinical outcomes of IVM embryos. Given the higher risk of meiotic abnormalities in IVM oocytes, euploidy screening is of particular importance [40]. However, previous studies have primarily focused on comparing the aneuploidy rates between IVM and IVF embryos, with inconsistent conclusions. For instance, Li et al. reported no significant difference in the aneuploidy rate between IVM embryos and in vivo‐matured counterparts [41], whereas a study on rescue‐IVM embryos indicates a higher risk of chromosomal abnormalities [42]. Notably, few studies have specifically explored the relationship between the morphokinetic parameters of IVM embryos and chromosomal euploidy. Against this backdrop, our study is the first to identify tSB–t9 as a potential marker of chromosomal euploidy (*p* = 0.044), providing a new basis for assessing chromosomal stability in IVM‐derived embryos.

The median tSB–t9 for euploid blastocysts (24.0 h) was significantly shorter than for aneuploidy (35.7 h), indicating that the rapid initiation of blastocyst formation after the 9‐cell stage may be an important characteristic of chromosomally normal embryos. The mechanism may relate to the higher efficiency of DNA damage repair in euploid embryos, enabling rapid initiation of blastocyst formation (tSB) and expansion (tB) after the 9‐cell stage, while aneuploid embryos may activate stress pathways (e.g., p53‐mediated cell cycle arrest) due to chromosomal copy number abnormalities, leading to delayed blastocyst formation [43]. Notably, the predictive value of tSB–t9 remained significant in multivariate analysis (OR = 1.17), suggesting its potential as a complementary tool for PGT. In clinical practice, for IVM cycles where PGT is not feasible due to cost or embryo quality limitations, embryos with tSB–t9 ≤ 24 h may be prioritized, potentially improving implantation success rates.

An intriguing and clinically relevant observation emerged from our analysis of the t9–t7 interval. While a shorter duration was associated with a higher likelihood of blastocyst formation, among those blastocysts that formed, a longer t9–t7 interval was observed in euploid compared to aneuploid embryos. This apparent paradox may reflect a fundamental developmental speed–quality trade‐off pertinent to IVM‐derived embryos. The 7–9 cell stage coincides with major zygotic genome activation and the transition to embryonic control. A shorter interval here likely signifies robust transcriptional and metabolic activity, providing the developmental momentum necessary to overcome compaction and blastulation checkpoints. Conversely, a slightly prolonged passage through this critical window in euploid blastocysts might indicate more stringent cell‐cycle regulation, allowing time for adequate DNA repair and faithful chromosome segregation—factors crucial for genomic integrity but potentially at the cost of developmental pace. This complexity underscores that no single morphokinetic parameter is universally optimal; rather, it highlights the need for an integrated assessment. In clinical practice, t9–t7 may be most useful as a preliminary filter for developmental competence, while the selection of euploid embryos should heavily weight later‐stage predictors like tSB–t9 alongside comprehensive morphology. We acknowledge that the association between longer t9–t7 and euploidy requires cautious interpretation due to our limited sample size, and this hypothesis‐generating observation merits rigorous validation in larger IVM cohorts.

### 4.1 Strengths and Limitations

The strengths of this study lie in its use of advanced TLI technology, allowing real‐time tracking of IVM embryo development and precise quantification of key morphokinetic parameters. This approach provides valuable insights into IVM embryo development and improves embryo selection strategies in ART. Additionally, by focusing specifically on IVM embryos, we addressed a gap in the literature, as most prior research has centered on conventional IVF/ICSI embryos. Furthermore, this study integrates key clinical outcomes, namely blastocyst formation and chromosomal normality, into a comprehensive morphokinetic screening approach with high clinical application value. However, this study also has limitations. First, the relatively small sample size may limit the generalizability of the findings. Second, due to the limited sample size, we were unable to stratify the analysis by the initial maturation state (GV vs. MI) of the oocytes, which prevented us from accounting for this potential source of biological heterogeneity and confounding in the analysis. Furthermore, all morphokinetic measurements were standardized relative to the time of ICSI, ensuring that variations in the preceding IVM culture duration did not affect the comparability of developmental timing between embryos. A further limitation is the absence of clinical outcome data, such as pregnancy and live birth rates, following embryo transfer. Therefore, future studies are needed to directly correlate morphokinetic parameters with long‐term clinical endpoints.

## 5 Conclusions

This study systematically elucidates the morphokinetic characteristics of IVM‐derived embryos and demonstrates that S2 and t9–t7 are independent factors associated with blastocyst formation, while tSB–t9 is an effective marker for chromosomal euploidy in our preliminary analysis. These findings not only deepen our understanding of IVM embryo development but also provide reliable tools for non‐invasive screening of IVM embryos in clinical practice, offering the potential to expand the use of IVM technology in ART.

## Funding

This work was supported by the National Key Research and Development Program of China (No. 2024YFC2706701) and Tianjin Health Research Project (No. TJWJ2023MS028).

## Ethics Statement

The study was approved by the Ethics Committee of Tongji Hospital, Tongji Medical College, Huazhong University of Science and Technology (TJ‐IRB20211280).

## Conflicts of Interest

The authors declare no conflicts of interest.

## Supporting information


**Data S1:** rmb270037‐sup‐0001‐DataS1.docx.

## Data Availability

The data that support the findings of this study are available from the corresponding author upon reasonable request.
