# Supplementary material for: Morphokinetic Markers of Blastocyst Formation and Chromosomal Euploidy in Embryos Derived From In Vitro Maturation Oocytes
Source: Reprod Med Biol. 2026 Mar 30;25(1):e70037. doi: 10.1002/rmb2.70037 (PMC13045424; doi:10.1002/rmb2.70037)
Supplement: Supplementary file 1 — Data S1: rmb270037‐sup‐0001‐DataS1.docx. [file RMB2-25-e70037-s001.docx]

| **Additional Table 1.** Comparison of morphokinetic parameters between blastocyst formation and non-formation groups | | | |
| --- | --- | --- | --- |
| Parameters(h) | Blastocyst formation  (n=46) | Non-blastocyst formation  (n=37) | P value |
| tPNa | 7.2 (6.2-8.0) | 7.2 (6.6-9.5) | 0.213 |
| tPNf | 23.1 (21.7-26.2) | 25.5 (22.9-29.2) | 0.088 |
| t2 | 25.6 (24.0-29.1) | 28.2 (26.1-32.9) | 0.024^*^ |
| t3 | 35.4 (32.2-39.8) | 33.3 (30.4-40.5) | 0.735 |
| t4 | 36.8 (34.6-40.1) | 40.1 (33.3-43.7) | 0.211 |
| t5 | 46.7 (39.6-52.9) | 43.2 (37.2-51.6) | 0.216 |
| t6 | 50.7 (43.7-57.9) | 49.0 (43.1-57.6) | 0.472 |
| t7 | 54.3 (48.6-60.2) | 55.7 (47.9-68.8) | 0.438 |
| t8 | 58.9 (52.8-71.8) | 64.8 (55.3-74.4) | 0.251 |
| t9 | 70.0 (63.6-79.4) | 81.5 (67.9-95.4) | 0.024^*^ |
| VP | 15.8 (14.4-19.3) | 17.8 (14.8-20.7) | 0.241 |
| CC2 | 10.4 (9.2-12.1) | 9.8 (0.9-11.8) | 0.087 |
| CC3 | 11.9 (7.6-14.3) | 9.6 (1.2-15.2) | 0.254 |
| S2 | 0.5 (0.2-1.5) | 0.7 (0.3-8.3) | 0.050^*^ |
| S3 | 14.3 (4.5-20.1) | 17.8 (12.4-28.2) | 0.051 |
| t8-t7 | 2.0 (0.7-8.0) | 3.5 (1.5-13.4) | 0.106 |
| t9-t7 | 17.4 (11.2-20.1) | 20.9 (14.6-32.0) | 0.012^*^ |
| t9-t8 | 10.5 (2.3-17.6) | 14.1 (8.8-22.9) | 0.039^*^ |
| *Note:* Parameters were described as median (interquartile range) and compared using the Mann-Whitney U test.  The exact *P*-value for S2 is 0.049957, the value 0.050 shown reflects rounding to three decimal places. | | | |
| h, hour; tPNa, time to pronuclear appearance; tPNf, time to pronuclear fading; tn (n=2-9): time to n cells; VP, tPNf-tPNa; CC2, t3-t2; CC3, t5-t3; S2, t4-t3; S3, t8-t5. | | | |
| ^*^*P* < .05 |  |  |  |

| **Additional** **Table 2.** Comparison of morphokinetic parameters between euploid and aneuploid blastocysts groups | | | |
| --- | --- | --- | --- |
| Parameters(h) | Euploid blastocysts  (n=14) | Aneuploid blastocysts  (n=7) | P value |
| tPNa | 6.9 (6.2-8.0) | 7.4 (6.3-8.6) | 0.709 |
| tPNf | 23.1 (21.6-26.2) | 21.9 (19.7-25.9) | 0.412 |
| t2 | 25.6 (23.9-28.2) | 24.1 (22.4-29.0) | 0.370 |
| t3 | 35.9 (30.0-40.5) | 34.4 (31.6-36.0) | 0.526 |
| t4 | 36.1 (34.6-40.6) | 36.6 (32.4-39.6) | 0.576 |
| t5 | 47.5 (37.0-52.8) | 45.4 (42.5-49.6) | 0.709 |
| t6 | 49.5 (42.1-56.2) | 50.1 (43.8-51.0) | 0.881 |
| t7 | 52.6 (47.3-57.4) | 53.8 (46.3-54.5) | 0.765 |
| t8 | 55.7 (49.3-65.7) | 55.1 (49.2-67.3) | 0.881 |
| t9 | 69.4 (64.1-80.2) | 67.8 (57.9-74.3) | 0.218 |
| tSB | 93.6 (85.0-99.9) | 95.7 (91.9-109.7) | 0.247 |
| tB | 101.6 (95.4-112.6) | 105.6 (104.6-117.8) | 0.086 |
| VP | 16.0 (14.5-19.5) | 14.9 (13.4-18.5) | 0.314 |
| CC2 | 10.8 (7.8-12.2) | 10.3 (9.4-10.9) | 0.478 |
| CC3 | 11.9 (10.1-13.6) | 13.0 (8.8-14.0) | 0.852 |
| S2 | 0.3 (0.2-2.3) | 0.4 (0.2-1.7) | 0.572 |
| S3 | 9.3 (1.4-20.2) | 14.8 (3.8-18.9) | 0.681 |
| t8-t7 | 1.1 (0.7-7.1) | 2.9 (0.2-14.1) | 0.681 |
| t9-t7 | 20.0 (14.9-21.2) | 15.2 (11.6-17.2) | 0.044^*^ |
| t9-t8 | 15.3 (5.4-18.1) | 2.2 (1.1-15.0) | 0.156 |
| tSB-t7 | 40.7 (34.4-46.3) | 43.5 (40.7-55.9) | 0.179 |
| tSB-t8 | 36.2 (31.8-40.1) | 43.2 (26.3-44.4) | 0.205 |
| tSB-t9 | 24.0 (15.9-27.7) | 35.7 (24.1-41.9) | 0.025^*^ |
| tB-t7 | 49.0 (42.5-57.5) | 57.3 (53.4-64.0) | 0.073 |
| tB-t8 | 46.8 (40.5-50.4) | 53.3 (37.3-56.3) | 0.156 |
| tB-t9 | 32.6 (23.6-37.5) | 46.7 (36.2-56.1) | 0.014^*^ |
| tB-tSB | 8.9 (6.7-11.6) | 10.6 (8.1-12.7) | 0.332 |
| *Note:* Parameters were described as median (interquartile range) and compared using the Mann-Whitney U test. | | | |
| h, hour; tPNa, time to pronuclear appearance; tPNf, time to pronuclear fading; tn (n=2-9): time to n cells; tSB, time to start of blastulation; tB, time to blastocyst formation; VP, tPNf-tPNa; CC2, t3-t2; CC3, t5-t3; S2, t4-t3; S3, t8-t5. | | | |
| ^*^*P* < .05 |  |  |  |

| **Additional Table 3.** Mixed-effects logistic regression analysis for blastocyst formation | | |
| --- | --- | --- |
| Parameters (h) | P value | OR (95%CI) |
| S2 | 0.035^*^ | 1.16 (1.01-1.33) |
| t9-t7 | 0.012^*^ | 1.09 (1.02-1.17) |
| h, hour; S2, t4-t3. | | |
| ^*^*P* < .05 | | |
